# Randomized Trial of Early Enhanced Parenteral Nutrition and Later Neurodevelopment in Preterm Infants

**DOI:** 10.3390/nu14193890

**Published:** 2022-09-20

**Authors:** Erin E. Morris, Neely C. Miller, Nicholas A. Marka, Jennifer L. Super, Emily M. Nagel, Juan David Gonzalez, Ellen W. Demerath, Sara E. Ramel

**Affiliations:** 1 Department of Pediatrics, Division of Neonatology, University of Minnesota, Minneapolis, MN 55454, USA; 2 Masonic Institute for the Developing Brain, University of Minnesota, Minneapolis, MN 55414, USA; 3 Clinical and Translational Science Institute, University of Minnesota, Minneapolis, MN 55455, USA; 4 Division of Epidemiology and Community Health, School of Public Health, University of Minnesota, Minneapolis, MN 55454, USA; 5 Neonatology, Children’s Minnesota, Minneapolis, MN 55404, USA

**Keywords:** prematurity, enhanced nutrition, parenteral nutrition, enteral nutrition, neurodevelopment

## Abstract

Retrospective studies indicate that the parenteral provision of calories, proteins, and lipids in the first week of life is associated with improved later neurodevelopment. We aimed to determine whether infants randomized to an enhanced parenteral nutrition protocol had improved developmental outcomes at 4, 12, or 24 months corrected age (CA). In total, 90 preterm infants (<32 weeks gestational age and <1500 g) were randomized to receive enhanced parenteral nutrition (PN) or standard PN during the first week of life. The enhanced group received a higher glucose infusion rate and intralipids. Neurodevelopmental outcomes included pattern-reversal visually evoked potentials (VEP) at 4 months CA (*n* = 33) and the Bayley Scales of Infant Development (BSID) at 12 (*n* = 46) and 24 (*n* = 29) months CA. P100 latency was longer in the intervention group, indicating slower processing speed (145 vs. 178 ms, *p* = 0.01). This association did not hold in multivariable analysis adjusting for potentially confounding variables. BSID scores were not associated with enhanced PN. Higher enteral energy and protein intake regardless of randomization group were associated with faster processing speed at 4 months CA (*p* ≤ 0.02 for both). Enhanced early PN was not associated with improved neurodevelopment; however, first-week enteral caloric and protein intake were associated with improved speed of processing.

## 1. Introduction

For infants born prematurely and at very low birth weight (VLBW), nutrition is an important predictor of growth and neurodevelopmental outcomes. While many clinical factors contributing to neurodevelopment are not modifiable, optimal early nutrition provides an avenue for improving neurodevelopmental outcomes in this vulnerable population. Due to clinical illness and a limited feeding tolerance, a significant portion of nutrition during the first week of life is given parenterally. Despite the importance of early nutrition, the optimal provision of calories and specific macronutrient intake for growth and neurodevelopment in very preterm infants is not yet clear [1,2,3].

Several studies support the positive impact of early parenteral nutrition (PN) on preterm infants’ weight, length, and head circumference [4,5,6]. In conjunction, observational studies linked this early growth with later neurodevelopment in both childhood [7] and till adulthood [8,9]. Our group consistently found a positive association between early fat-free mass gains and later neurodevelopmental outcomes [10,11].

Despite this body of the literature suggesting the positive neurodevelopmental effects of enhanced nutrition, recent studies found higher rates of neurodevelopmental impairment and lower developmental scores for preterm infants receiving enhanced early parenteral nutrition [12,13]. A systematic review by De Nardo et al. also recommended a cautious approach to enhanced nutritional strategies due to these neurodevelopmental concerns [14], leaving the optimal PN strategy to be determined.

In addition to the lack of clarity in neurodevelopmental outcomes, the majority of studies focused on early nutrition were not randomized and thus risked confounding by non-nutritional factors, especially degree of illness. There is also large variation in the nutritional approach, and reported intakes and outcomes. With this paucity of data, our group was interested in the effects of early enhanced parenteral nutrition on later neurodevelopment in very preterm infants. The objective of this study was to determine whether infants randomized to an enhanced parenteral nutrition protocol vs. a standard nutrition protocol in the first week of life had improved speed of processing at 4 months corrected age (CA) or improved performance on the Bayley Scales of Infant Development (BSID) at 12 or 24 months CA.

## 2. Materials and Methods

### 2.1. Study Design

A total of 90 very low birth weight (VLBW) preterm infants were enrolled and randomized to this partially blinded nutrition intervention trial between August 2017 and June 2019 at the University of Minnesota Masonic Children’s Hospital Neonatal Intensive Care Unit (NICU). Infants were eligible if the gestational age (GA) was <32 weeks and birth weight <1500 g, and if the study team was able to obtain consent from the parents or guardians within 12 h of birth.

Infants were excluded if there was a prenatally diagnosed condition (other than prematurity) that affects growth, adiposity, or neurocognitive development; severe birth asphyxia; enrollment in another study affecting nutritional management; likely transfer out of NICU; and/or the inability of the family to follow-up after discharge.

The primary outcome of this randomized intervention trial was the feasibility of providing increased calories in the first week of life to VLBW preterm infants. These results were published elsewhere. The secondary outcomes of this trial, which are the focus of this manuscript, were the speed of processing at 4 months corrected age (CA) and performance on the Bayley Scales of Infant Development (BSID) at 12 and 24 months CA.

The study was approved by the Institutional Review Board of University of Minnesota (#00000063, 21 June 2017).

### 2.2. Study Population

**Figure 1:**
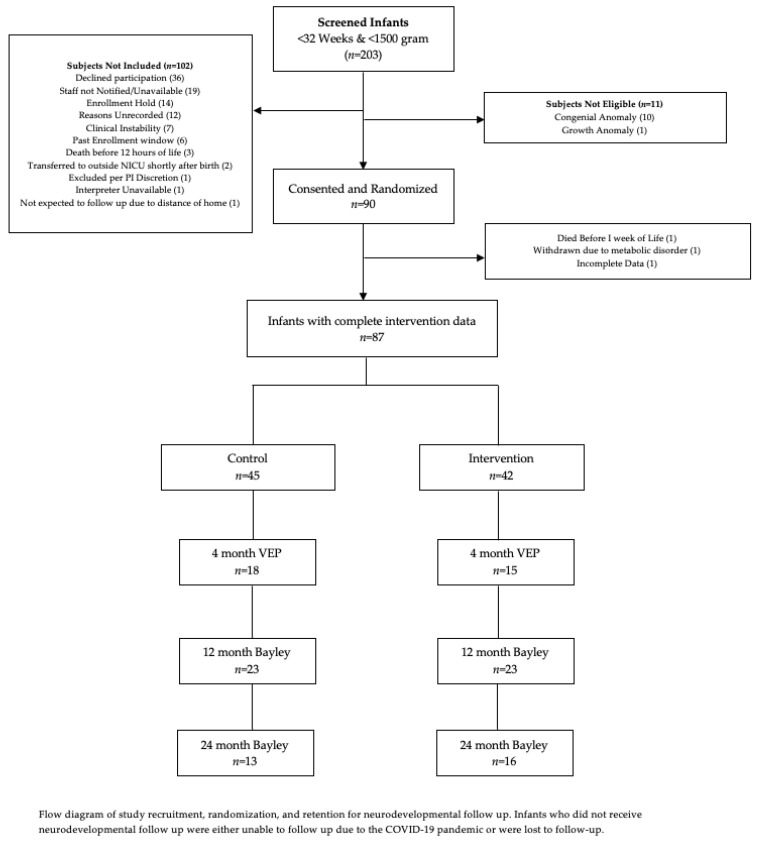
Flow diagram of study participation.

During the recruitment period, a total of 203 infants meeting the gestational-age and birth-weight criteria were admitted to the NICU. We enrolled 90 infants, of which 87 had complete intervention data. Recruitment numbers are detailed in a flow diagram in Figure 1.

The target enrolment of 90 infants for this pilot study was based on a goal of 45 infants in each treatment group (30 infants per GA stratum). Given that this was a pilot/feasibility trial, we were unable to perform formal power analysis. However, our numbers were chosen on the basis of findings from our previous observational trials.

### 2.3. Intervention

Enrolled infants were stratified by gestational-age group on the basis of the degree of prematurity (22–25, 26–29, 30–32 weeks) and then randomized to the enhanced nutrition protocol (intervention group) or standard parenteral nutrition protocol (control group) using the REDCap database. Within each GA stratum, permuted block randomization was used to assign infants to either the intervention group or the control group while preventing study personnel from being able to predict treatment allocation. The inpatient study coordinator, dietitian, and data analyst were unblinded to each infant’s randomization group, while investigators, study personnel, and parents were blinded to the randomization group.

Details of the intervention protocol are provided in Supplementary Table S1. Infants randomized to the intervention group followed an enhanced nutrition protocol consisting of starter total parenteral nutrition (TPN) initiated at 80 mL/kg/day to provide protein at 4 g/kg/day and a glucose infusion rate (GIR) of ~5.5 mg/kg/min. Custom TPN was started on DOL 2. GIR was advanced by ~1.5 mg/kg/min per day throughout the first week of life to a maximum of 12–14 mg/kg/min. Amino acids were maintained at 4 g/kg/day until adequate feeding volumes had been reached. Intralipids (ILs) were started at 2 g/kg/day and increased to 3.5 g/kg/day by DOL 3.

Infants randomized to the standard group received starter TPN at 60 mL/kg/day to provide 3 g/kg/day of protein and a GIR of ~4 mg/kg/min. ILs were provided at 0.5–1 g/kg/day. Custom TPN was started on DOL 2. GIR was advanced by 1 mg/kg/min to a maximum of 12–14 mg/kg/min and IL by 1 g/kg/day to a maximum of 3.5 g/kg/day by DOL 4 per standard protocol in our NICU. Protein was advanced to 4 g/kg/day on DOL 2 and remained at this rate until adequate feeding volumes had been reached.

Nutritional management was jointly agreed upon by the neonatologist and the neonatal dietitian. In circumstances of severe hypoglycemia, hyperglycemia, hypertriglyceridemia, or cholestasis in the first week of life, or when deemed to be in the best interest of the patient, deviation from the protocol occurred. All enteral feeding decisions and parenteral nutrition support after the first week of life were per the clinical discretion of the medical care team. Total fluid goals were at the discretion of the clinical team other than the rate of starter TPN on DOL 1.

Data collection included total calories (kcal/kg/day), protein (g/kg/day), and lipids (g/kg/day). The infants’ first week of intake was defined as the total number of kcal/kg/day and g/kg/day of protein received on days of life (DOL) 2–8 because of the variable time of birth and thus time on parenteral nutrition on DOL 1. Data were entered into REDCap, a secure web-based and password-protected database for storage, retrieval, and analysis.

### 2.4. Outpatient Neurodevelopmental Follow-Up

Visually evoked potentials (VEPs) were recorded at the 4 month corrected age (CA) follow-up visit. Data were included in this analysis if the visit had occurred between 3 and 7 months CA, and postmenstrual age was included as a covariate in the adjusted models to account for this variation. Bayley Scales of Infant Development (BSID) were collected at 12 and 24 months CA. Since the BSID accounts for the infant’s age at time of test, data were included in this analysis if the visit had occurred between 10 and 21 months CA for the 12-month BSID, and 18 to 32 months CA for the 24-month BSID.

At 4 months, infants had a 64-electrode Sensor Net (Electrical Geodesics, Inc., Eugene, OR, USA) placed on their head. The infants were then seated in a darkened room on their parent’s lap and presented with a visual stimulus of a pattern-reversing black-and-white checkerboard by E-Prime software version 2.0.8.90 (Psychology Software Tools, Sharpsburg, PA, USA). The pattern comprised ~1.5 cm × 1.5 cm individual checks (1.4 × 1.4°) with two reversals per second for 50 trials. A research assistant hidden behind the monitor and a cloth screen provided redirection to keep the participant’s attention on the monitor if needed [15]. Pattern-reversal VEP data were collected and recorded from the ongoing EEG using a Geodesic EEG System 200 (Electrical Geodesics, Inc. (EGI), Eugene, OR, USA), and NetStation 4.4.2 software (EGI) was used to measure scalp impedances (accepted if <50 kΩ) and process the data (referenced to the vertex, amplified with a 0.1–100 Hz bandpass and digitized at 250 Hz).

Using NetStation 4.4.2 analytical software (EGI), EEG data were filtered with a 30 Hz low-pass filter segmented to 500 ms periods starting 100 ms before the stimulus presentation and baseline corrected to the average prestimulus voltage. Segments were then hand-edited for movement artifact, eye movement, and poor recordings. As per the center protocol [16], trials were excluded if the number of rejected electrodes was >16%, and participants with <12 accepted trials (out of 50) were excluded from further analysis (mean number of accepted trials at 4 months was 38). For the accepted trials, bad data were replaced using a spherical spline interpolation, and the average waveform at each electrode was calculated and re-referenced to the average reference. The latency of the P100 component was measured by averaging Leads O1 and O2 to approximate Oz (not present on a 64 channel net). If an individual electrode did not have an identifiable P100, the data were treated as missing.

Neurodevelopmental testing using the Bayley Scales of Infant Development-III (BSID) was performed by a pediatric neuropsychologist at 12 and 24 months CA in the NICU follow-up clinic.

### 2.5. Data Analysis

Infant demographics are summarized as means and standard deviations for continuous variables, and frequencies and percentages for categorical variables. Univariate comparisons between study arms were conducted using *t*-tests for continuous outcomes and the chi-squared or Fisher’s exact test for categorical outcomes as appropriate. Linear regression models were used to assess univariate and multivariable adjusted associations for the VEP and BSID developmental outcomes. All analyses were conducted at the 0.05 significance level using R statistical software version 4.1.0 [17].

Sensitivity analysis was conducted to determine the impact attrition bias on the multivariable model results. Using infant demographic and clinical covariates, a logistic regression model for the probability of being included (versus missing) at the time of outcome assessment was utilized. For each of the missing probability models, predictors were selected using variables associated with both missingness and the outcome of interest [18]. Using the inverse of the predicted probability of being included, weighted multivariable linear regressions as described above were ran. Potential attrition bias was noted in any instances where the significance decision changed from the intention to treat analysis, and where the effect estimate direction and magnitude differed significantly.

## 3. Results

Of the 90 infants enrolled in the study, 33 had a VEP follow-up, 46 had BSID at 12 months CGA, and 29 infants had BSID at 24 months CGA. Study recruitment occurred between August 2017 and June 2019, and neurodevelopmental follow-up extended through April 2022. The study concluded when the target enrollment of 90 infants had been reached. A flow diagram of study participation is provided in Figure 1.

Baseline demographics and inpatient variables for the entire cohort were similar (Table 1). Analysis of the baseline demographics in the neurodevelopmental follow-up group revealed a difference in Hispanic/Latino ethnicity within the VEP group (control: 4 infants (22%); intervention: 0 infants (0%), *p* = 0.018). Additionally, there were more antibiotic days in the first week for the intervention group subjects with VEP and 12-month BSID follow-up (*p* ≤ 0.03). Analysis based on infants lost to neurodevelopmental follow-up is also provided in Supplementary Table S2. Demographics of those lost to follow-up were similar with the exception that those lost to follow-up for 12-month CA BSID were more likely to be born to younger, non-White mothers.

As expected, average caloric intake (total enteral + parenteral) over Days 2–8 was statistically different for the entire cohort, with an average intake of 375.3 kJ/kg/day (89.7 kcal/kg/day (SD 16.1)) in the control group, and 429.3 kJ/kg/day (102.6 kcal/kg/day (SD 12.5)), *p* < 0.001. When there were multiple neurodevelopment follow-up groups, this difference was lost for the 24-month Bayley group (Table 2). Parenteral intake was higher in the intervention group for infants in all follow-up groups. However, the average enteral and protein intake over Days 2–8 was higher in the control group for infants in the 4-month VEP and 12-month BSID groups. Age analysis at starting enteral feeds, and caloric and protein deficits across the hospital stay were not different for the entire cohort or the neurodevelopmental follow-up groups (Table 1 and Supplementary Table S2).

In this trial, infants who had received enhanced PN did so without increased metabolic complications such as hyperglycemia, hypertriglyceridemia, hyperbilirubinemia, or increased days of insulin use when compared to the control group. These results, and the safety and feasibility of the study protocol, were presented separately by our group [19].

Univariate intention to treat the analysis of VEP data showed longer P100 latency in the intervention group, suggestive of slower speed of processing (control 145 vs. intervention 177 ms, *p* = 0.01). BSID outcomes were similar at 12 and 24 months CA (Table 3). Multivariable analysis found a similarly longer P100 latency in the intervention group in adjusted Model 1 (for sex, gestational age, and age at VEP), but the difference disappeared in Model 2, which was additionally adjusted for calorie intake from enteral nutrition (Table 4).

These data were also analyzed by caloric and protein intake (total enteral + parenteral and enteral alone) in the first week of life regardless of randomization group. To assess whether associations with speed of processing or BSID outcomes were specific to first-week caloric intake, multivariable analyses were conducted (adjusting for sex, gestational age and age at VEP). Results show an association between enteral caloric intake and P100 latency, such that, for every enteral energy intake increase of 42 kJ/kg/day (10 kcal/kg/day), there was a 10.13 ms faster speed of processing at 4-month VEP (effect estimate −1.13 (−2.011, −0.26) *p* = 0.013). Additionally, for every increase in enteral protein of 1 g/kg/day, there was 29.34 ms faster speed of processing at 4-month VEP (effect estimate −29.34 (−57.89, −0.8) *p* = 0.044). In multivariable analysis, there were no associations between total enteral caloric or protein intake and BSID outcomes. Additionally, there were no associations between total caloric (parenteral + enteral) intake and speed of processing. The multivariable analysis of parenteral intake alone revealed that, for every 42 kJ/kg/day (10 kcal/kg/day) increase, there was a 9.4 ms reduction in speed of processing (*p* = 0.036), and for every g/kg/day parenteral protein, there was a 52.33 ms reduction in speed of processing (*p* = 0.027).

Due to the high percentage lost to follow-up in this cohort during the COVID-19 pandemic, sensitivity analysis for attrition bias using inverse probability weighting was completed and confirmed the original findings. We found slower speed of processing in the intervention group in Model 1 (effect estimate 34.61 (11.97, 57.25) *p* = 0.004), and the difference again disappeared in Model 2, which adjusted for first-week enteral nutrition (effect estimate 22.43 (−4.81, 49.68) *p* = 0.103) (Table 5). Effect estimates were similar, and confidence intervals were similar or narrowed compared to initial models. Sensitivity analysis based on caloric and protein intake regardless of intervention group also reflected the findings in our initial analysis. The details and results of this sensitivity analysis are provided in Supplementary Table S2.

## 4. Discussion

In this randomized trial, enhanced early PN during the first week of life did not improve the speed of processing at 4-month CA or developmental scores at 12 and 24 months CA. In contrast, we found slower speed of processing in the group randomized to enhanced PN, and no difference in BSID scores between the groups. Parenteral caloric and protein intake regardless of randomization group were associated with slower speed of processing at 4-month CA, while enteral caloric and protein intake were associated with faster speed of processing. The differences in the speed of processing between randomization groups were no longer statistically different after adjusting for enteral intake in the first week.

Our analysis was complicated by a higher proportion of enteral feedings in the control-group subjects that returned for neurodevelopmental follow-up. This may indicate a healthier group of infants in the control group that returned for follow-up, as the timing of the initiation and proportion of enteral feedings was based on individual clinician discretion. The only other significant differences in illness severity was more antibiotic days in the intervention group who had returned for VEP and 12-month BSID follow-up. Despite these differences in the follow-up groups, our findings suggest enhanced parenteral nutrition should be given with caution until larger randomized trials have been completed.

The relationship between enhanced early PN and slower speed of processing complements two recent studies urging caution regarding the potential negative consequences of enhanced early PN. In a cohort study of 245 infants by Barreault et al. [12], there was a higher likelihood of neurodevelopmental impairment (cerebral palsy diagnosis or ages and stages questionnaire score < 2 standard deviations below the mean) for infants with higher first-week protein intake or second-week nonprotein energy intake. These nutritional intakes included both parenteral and enteral nutrition, with mean protein intake of 3.45 g/kg/day in the first week, and mean caloric intake of 431 kJ/kg/day (103 kcal)/kg/day in the second week. However, this was not a randomized trial; therefore, infants receiving more parenteral nutrition may have had other risk factors for poorer outcomes.

A second cohort study of 51 preterm infants (GA < 32 weeks) by Terrin et al. [13] investigated enhanced PN in the first 7 days of life. Specifically, they studied differences in outcomes between an energy-enhanced and an energy-standard group that differed in first-week lipid and dextrose intake, but not in protein intake, similar to the intervention in this study. The enhanced group had lower motor scores and socioemotional competence performance on Bayley developmental testing at 24-month CA. In conjunction with our study, these findings suggest that increased parenteral energy intake may independently add risk to neurodevelopment.

Conversely, others have shown improved development with enhanced PN. In a study by Stephens et al., increased first-week protein and energy intake (of which 98% was parenteral) was associated with improved developmental scores at 18-month CA [20]. The study reported an increase of 4.6 points in the Mental Development Index for each additional 42 kJ/kg/day (10 kcal)/kg/day, and an 8.2 point increase for each additional gram/kg/day of protein provided. Other studies reported early enhanced PN to be associated with improved head circumference at 36-week CA [21], increased developmental quotient at 1-year CA [22], and decreased incidence of brain lesions on MRI [23].

Individual nutrient intake may have a more profound impact on neurodevelopment. Observational studies found early protein provision to be associated with larger brain volumes, higher language [24] and cognitive scores [25], and greater functional connectivity and greater processing speed [26]. However, randomized trials were less conclusive [27,28]. Lipids are also a major contributor to overall energy intake, and increased early intake was correlated with improved cerebellar volumes at term [29] and with improved developmental quotient at 1-year CA [22].

Another study investigating the association with enhanced nutrition and neurodevelopmental outcomes used earlier and more rapid advancement of enteral nutrition in conjunction with PN, and found improved language development at 18-month CA [24]. There is a paucity of studies that considered the effect of enteral and parenteral nutrition separately, and many studies that observed the positive effects of enhanced nutrition on cerebral and basal ganglia growth have only investigated the influence of enteral nutrition [14]. A study by Boscarino et al. examined the differential effects of enteral nutrition and PN on head growth, and found enteral nutrition to be associated with increased caudate and cerebellar vermis growth, while PN was independently associated with decreased growth in the caudate and cerebellar transverse diameter [30].

There are several physiologic mechanisms that may underlie the relationship between enhanced early PN and worse neurodevelopmental outcomes. First, while we did not find differences in hyperglycemia, direct hyperbilirubinemia, or hypertriglyceridemia among the groups, it is possible there are more subtle metabolic consequences of enhanced parenteral nutrition that may impact neurodevelopment and are not detected with intermittent monitoring [31,32]. Second, while protein provision was not part of our intervention, the protein/energy ratio is a crucial aspect of providing parenteral nutrition. The appropriate intake of each limits the intake of the other [33]. Additionally, the high-energy state induced by enhanced parenteral nutrition may induce microvascular dysfunction, altering tissue perfusion and compromising cerebral blood flow at a critical period of development [13,34].

Unfortunately, due to the COVID-19 pandemic and limitations on outpatient follow-up visits, our return rate was quite low and has limited the generalizability of the data. While this is a randomized trial, the benefit of randomization is jeopardized when follow-up rates are low. Additionally, while the follow-up groups were largely similar, the differences in enteral intake may suggest that the control group that returned for follow-up was more clinically stable in the first week of life. Sensitivity analysis for attrition bias, however, reinforces our original findings and improves the applicability of our results.

## 5. Conclusions

Infants randomized to enhanced early PN during the first week of life did not show improved speed of processing at 4-month CA, or BSID scores at 12- or 24-month CA. Analysis regardless of randomization group revealed parenteral nutrition (both caloric and protein intake) to be associated with slower speed of processing at 4-month CA and enteral nutrition (calorie and protein intake) to be associated with improved speed of processing. Our findings highlight the importance of early enteral nutrition to later neurodevelopment, and emphasize the need for further research before these findings can be generalized and before enhanced PN is routinely utilized in clinical settings. Larger randomized trials are needed to investigate the impact of early nutrition on later development and to determine optimal caloric intake goals for preterm neonates in the first week of life.

## Figures and Tables

**Table 1:** Infant demographics and baseline inpatient variables. Results displayed as mean (SD) or *n* (%).

	Control (*n* = 45)	Intervention (*n* = 42)	*p*-Value
Gestational age (weeks)	27.4 (2.6)	27 (2.4)	0.447
Birth weight (kg)	1 (0.3)	0.9 (0.3)	0.567
Sex (% male)	25 (55.6%)	19 (45.2%)	0.455
Race			0.406 *
Asian	4 (8.9%)	3 (7.1%)	
Black	2 (4.4%)	7 (16.7%)	
White	30 (66.7%)	26 (61.9%)	
More than one race	1 (2.2%)	1 (2.4%)	
Other	8 (17.8%)	5 (11.9%)	
Ethnicity Hispanic/Latino	7 (15.6%)	4 (9.5%)	0.643 *
Maternal age	30.8 (5.8)	30.6 (5.5)	0.899
Antenatal corticosteroids	38 (84.4%)	40 (95.2%)	0.111 *
SGA	2 (4.9%)	2 (4.9%)	>0.99 *
Twin gestation	16 (35.6%)	6 (14.3%)	0.042
Apgar score < 5 at 5 min	3 (6.7%)	5 (11.9%)	0.475
Antibiotic use (Days 1–7)	2.2 (1.7)	2.9 (1.9)	0.475
SNAPPE II score	24.3 (26.8)	25.8 (19.6)	0.773
NEC	3 (6.8%)	4 (9.5%)	0.71 *
Chronic lung disease at 36 weeks	26 (60.5%)	26 (68.4%)	0.608
IVH (Grade 2+)	8 (17.8%)	6 (14.3%)	0.88
ROP (Stage 2/3)	8 (19%)	9 (23.7%)	0.816
Age at VEP (corrected age in days)	124.6 (16.6)	124.1 (14.4)	0.927
Mean parenteral kcal intake Days 2–8 (kcals/kg/day)	65.2 (10.5)	81.6 (11.1)	<0.001
Mean parenteral protein intake Days 2–8 (g/kg/day)	3.5 (0.5)	3.6 (0.4)	0.065
Mean day of enteral feed Initiation	3.1 (7.2)	1.8 (1.8)	0.298
Caloric deficit over stay (120 kcal/day goal)	201.8 (702.4)	16.1 (628.1)	0.197
Protein deficit over stay (4 g/kg/day goal)	−5 (24.8)	−7.6 (20.2)	0.596

* Fisher’s exact test instead of chi-squared test.

**Table 2:** Inpatient nutritional variables by neurodevelopmental follow-up group. Results displayed as mean (SD).

	Neurodevelopmental Test	Control	Intervention	*p*-Value
Average intake (**total enteral + parenteral**) Days 2–8 (kcal/kg/day)	VEP	**93.7 (13.7)**	**104.2 (10.1)**	**0.017**
	12-month Bayley	**91.3 (18.4)**	**100.8 (10.7)**	**0.04**
	24-month Bayley	92 (19.7)	101.3 (12.4)	0.154
Average **protein** intake (total enteral + parenteral) Days 2–8 (g/kg/day)	VEP	4.3 (0.3)	4.2 (0.3)	0.497
	12-month Bayley	4.1 (0.6)	4.1 (0.2)	0.932
	24-month Bayley	4.2 (0.3)	4.2 (0.3)	0.551
Average **enteral** intake, Days 2–8 (kcal/kg/day)	VEP	**31.4 (18.1)**	**17.5 (14.2)**	**0.019**
	12-month Bayley	**26.2 (19)**	**14.7 (14.2)**	**0.024**
	24-month Bayley	25.1 (19.1)	16.5 (16.1)	0.207
Average **enteral protein** intake, Days 2–8 (g/kg/day)	VEP	**0.9 (0.7)**	**0.4 (0.4)**	**0.022**
	12-month Bayley	**0.8 (0.7)**	**0.4 (0.5)**	**0.047**
	24-month Bayley	0.7 (0.8)	0.5 (0.6)	0.323
Average **parenteral** intake, Days 2–8 (kcal/kg/day)	VEP	**62.3 (9)**	**86.7 (8.9)**	**<0.001**
	12-month Bayley	**65.1 (11.3)**	**86.1 (8.9)**	**<0.001**
	24-month Bayley	**66.9 (12.9)**	**84.8 (10.2)**	**<0.001**
Average **parenteral protein** intake Days 2–8 (g/kg/day)	VEP	**3.4 (0.3)**	**3.8 (0.2)**	**0.002**
	12-month Bayley	**3.4 (0.5)**	**3.8 (0.3)**	**0.009**
	24-month Bayley	3.6 (0.4)	3.8 (0.3)	0.163

Sample size per neurodevelopmental test group: VEP *n* = 18 (control) *n* = 15 (intervention); 12-month Bayley *n* = 23 (control) *n* = 23 (intervention); 24-month Bayley *n* = 13 (control) *n* = 16 (intervention).

**Table 3:** Neurodevelopmental outcomes by randomization group—univariate analysis. Results displayed as mean (SD).

	Control	Intervention	*p*-Value
**Visually Evoked Potential**	*n* = 18	*n* = 15	
P100 Latency	**145.3 (32.6)** **ms**	**177.8 (34.7)** **ms**	**0.01**
**Bayley—12-month CGA**	*n* = 23	*n* = 23	
Cognitive	94.9 (18)	98.4 (16.6)	0.496
Language	85.5 (19.6)	87.6 (18.4)	0.712
Motor	86.5 (18.1)	87.3 (22.1)	0.885
**Bayley—24-month CGA**	*n* = 13	*n* = 16	
Cognitive	92.3 (22.8)	96.3 (14.9)	0.593
Language	94.8 (22.1)	95.5 (18.2)	0.933
Motor	89.2 (20.4)	88.1 (19.9)	0.884

**Table 4:** Neurodevelopmental outcomes by randomization group—multivariable adjusted models.

Neurodevelopmental Test	Model 1 Adjusted for Sex, Gestational Age, and Age at VEP			Model 2 Adjusted for Sex, Gestational Age, kcal/kg from Enteral Feedings, and Age at VEP		
	Effect Estimate	95% CI	*p*-Value	Effect Estimate	95% CI	*p*-Value
**VEP—P100 Latency** Control (*n* = 18) Intervention (*n* = 15)						
	**32.89**	**(7.98, 57.79)**	**0.012**	22.81	(−4.5, 50.13)	0.098
**Bayley—12-month CGA** Control (*n* = 23) Intervention (*n* = 23)						
Cognitive	4.72	(−4.33, 13.78)	0.298	4.46	(−5.54, 14.47)	0.373
Language	3.99	(−5.74, 13.73)	0.412	2.12	(−8.58, 12.82)	0.691
Motor	2.6	(−7.25, 12.44)	0.597	1.47	(−9.37, 12.31)	0.785
**Bayley—24-month CGA** Control (*n* = 13) Intervention (*n* = 16)						
Cognitive	1.16	(−12.99, 15.3)	0.867	2.73	(−13.56, 19.02)	0.732
Language	−0.19	(−15.61, 15.24)	0.98	3.4	(−14.12, 20.92)	0.691
Motor	−3.84	(−16.77, 9.08)	0.546	−3.49	(−18.4, 11.41)	0.633

**Table 5:** Neurodevelopmental outcomes by randomization group with sensitivity analysis for attrition bias.

Neurodevelopmental Test	Model 1 Adjusted for Sex, Gestational Age, and Age at VEP			Model 2 Adjusted for Sex, Gestational Age, Kcal/kg from Enteral Feedings, and Age at VEP		
	Effect Estimate	95% CI	*p*-Value	Effect Estimate	95% CI	*p*-Value
**VEP—P100 Latency** Control (*n* = 18) Intervention (*n* = 15)						
	**34.61**	**(11.97, 57.25)**	**0.004**	22.43	(−4.81, 49.68)	0.103
**Bayley—12-month CGA** Control (*n* = 23), intervention (*n* = 23)						
Cognitive	6.5	(−2.41, 15.42)	0.148	6.44	(−3.14, 16.03)	0.181
Language	3.14	(−6.17, 12.44)	0.5	1.52	(−8.4, 11.44)	0.758
Motor	3.47	(−6.36, 13.3)	0.479	2.09	(−8.39, 12.57)	0.689
**Bayley—24-month CGA** Control (*n* = 13), intervention (*n* = 16)						
Cognitive	0.54	(−14.64, 15.73)	0.942	2.13	(−15.43, 19.69)	0.804
Language	−1.33	(−17.39, 14.72)	0.865	1.77	(−16.64, 20.18)	0.844
Motor	−4.43	(−17.71, 8.85)	0.499	−4.15	(−19.51, 11.22)	0.583

## Data Availability

Data described in this manuscript are available in Supplementary Materials tables or available upon request.

## Supplementary Materials

The following supporting information can be downloaded at: https://www.mdpi.com/article/10.3390/nu14193890/s1. Table S1: Parenteral nutrition protocol during first week of life for the Intervention and Standard Groups, Table S2: Demographics & Inpatient Nutrition by Missing VEP Status, Table S3: Demographics & Inpatient Nutrition by Missing 1 Year Bayley Status, Table S4: Demographics & Inpatient Nutrition by Missing 2 Year Bayley Status, Table S5: ERP/VEP Analysis—Model 1 (Adjusted for Sex, Gestational Age, and Age at Test [VEP/ERP Only]), Table S6: ERP/VEP Analysis—Model 2 (Adjusted for Sex, Gestational Age, Kcal/kg from Feeds, and Age at Test [VEP/ERP Only]), Table S7: ERP/VEP Analysis—Model 3: Multivariable Condition by Specific Intake Days 2–8, Table S8: Bayley Score—Year 1—Model 1 (Adjusted for Sex, Gestational Age, and Age at Test [VEP/ERP Only]), Table S9: Bayley Score—Year 1—Model 2 (Adjusted for Sex, Gestational Age, Kcal/kg from Feeds, and Age at Test [VEP/ERP Only]), Table S10: Bayley Score—Year 1—Model 3—Multivariable Condition by Specific Intake Days 2–8, Table S11: Bayley Score—Year 2—Model 1 (Adjusted for Sex, Gestational Age, and Age at Test [VEP/ERP Only]), Table S12: Bayley Score—Year 2—Model 2 (Adjusted for Sex, Gestational Age, Kcal/kg from Feeds, and Age at Test [VEP/ERP Only]), Table S13: Bayley Score—Year 2—Model 3: Multivariable Condition by Specific Intake Days 2–8.
