# Supplementary material for: Randomized Trial of Early Enhanced Parenteral Nutrition and Later Neurodevelopment in Preterm Infants
Source: Nutrients. 2022 Sep 20;14(19):3890. doi: 10.3390/nu14193890 (PMC9570539; doi:10.3390/nu14193890)
Supplement: Supplementary file 1 [file nutrients-14-03890-s001.zip › nutrients-1873872-Supplementary.pdf]

nutrients

Supplementary Materials

Randomized Trial of Early Enhanced Parenteral Nutrition and Later Neurodevelopment in Preterm Infants

Erin E. Morris [1], Neely C. Miller [2], Nicholas A. Marka [3], Jennifer L. Super [1], Emily M. Nagel [4], Juan David Gonzalez [5], Ellen W. Demerath [4] and Sara E. Ramel [1,2,*]

Table S1. Parenteral nutrition protocol during first week of life for the Intervention and Standard Groups.

		Day 1	Day 2	Day 3	Day 4	Day 5	Day 6	Day 7
Intervention	GIR (mg/kg/min)	5.5	7	8.5	10	11.5	12	12
	IL (g/kg)	2	3	3.5	3.5	3.5	3.5	3.5
	Calories (kcal/kg)	47.5	55	77.5	85	92.5	95	95
Standard	GIR (mg/kg/min)	4.2	5	6	7	8	9	10
	IL (g/kg)	0.5	1.5	2.5	3.5	3.5	3.5	3.5
	Calories (kcal/kg)	26	40	55	70	75	80	85

Each g/kg of IL (Intralipid) provides 10 kcal/kg.
Each 1 mg/kg/min of GIR (Glucose Infusion Rate) provides ~5 kcal/kg.

Results of Inverse Probability Weighting and Sensitivity Analysis for Attrition Bias

Table S2. Demographics & Inpatient Nutrition by Missing VEP Status.

	**Missing (N = 54)**	**Present (N = 33)**	***p*-value**
Gestational Age [weeks]			0.417
Mean (SD)	27 (2.6)	27.5 (2.4)	
Birth Weight [kg]			0.736
Mean (SD)	0.9 (0.3)	1 (0.3)	
Sex			0.599
Female	25 (46.3%)	18 (54.5%)	
Male	29 (53.7%)	15 (45.5%)	
Race			0.384*
Asian	6 (11.1%)	1 (3%)	
Black/African American	7 (13%)	2 (6.1%)	
White/Caucasian	31 (57.4%)	25 (75.8%)	
More Than One Race	2 (3.7%)	0 (0%)	
Other/Unknown	8 (14.8%)	5 (15.2%)	
White Race Status			0.133
White	25 (75.8%)	31 (57.4%)	
Non-White	8 (24.2%)	23 (42.6%)	
Ethnicity			0.941*
Hispanic or Latino	7 (13%)	4 (12.1%)	
Not Hispanic or Latino	38 (70.4%)	22 (66.7%)	
Unknown/Not Reported	9 (16.7%)	7 (21.2%)	
Maternal Age [years]			> 0.99
Mean (SD)	30.7 (6.2)	30.7 (4.8)	
Antenatal Corticosteroid Use			0.048*
Yes	46 (85.2%)	32 (97%)	
No	7 (13%)	0 (0%)	
Unknown	1 (1.9%)	1 (3%)	
SGA			0.641*
Yes	2 (4%)	2 (6.2%)	
No	48 (96%)	30 (93.8%)	
Twin Status			0.668
Twin	15 (27.8%)	7 (21.2%)	
Not A Twin	39 (72.2%)	26 (78.8%)	
Apgar Score at 5 Min			0.353
Mean (SD)	6.9 (2)	7.2 (1.6)	
Antibiotic Use (Days 1–7) [days]			0.613
Mean (SD)	2 (2)	1.7 (1.8)	
SNAPPE II Scores			0.283
Mean (SD)	27 (25.9)	21.8 (18.8)	
Nec Status			0.703*
Yes	5 (9.4%)	2 (6.1%)	
No	48 (90.6%)	31 (93.9%)	
Chronic Lung Disease or BPD at 36 Weeks			0.747
Yes	32 (66.7%)	20 (60.6%)	
No	16 (33.3%)	13 (39.4%)	
IVH Status			0.233*

None/Grade 1	43 (79.6%)	30 (90.9%)	
Grade 2/3/4	11 (20.4%)	3 (9.1%)	
ROP Status			0.776
None/Stage 0/1	36 (76.6%)	27 (81.8%)	
Stage 2/3	11 (23.4%)	6 (18.2%)	
Average Intake (Total Enteral + Parenteral) Days 2–8 [kcal/kg/day]			0.213
Mean (SD)	94.4 (17.1)	98.5 (13.1)	
Average Protein Intake (Total Enteral + Parenteral) Days 2–8 [g/kg/day]			0.197
Mean (SD)	4.1 (0.4)	4.2 (0.3)	
Average Enteral Intake Days 2–8 [kcal/kg/day]			0.375
Mean (SD)	21.4 (19.4)	25 (17.6)	
Average Enteral Protein Intake Days 2–8 [g/kg/day]			0.9
Mean (SD)	0.7 (0.7)	0.7 (0.6)	
Average Parenteral Intake Days 2–8 [kcal/kg/day]			0.878
Mean (SD)	72.9 (12.7)	73.4 (15.2)	
Average Parenteral Protein Intake Days 2–8 [g/kg/day]			0.499
Mean (SD)	3.5 (0.5)	3.6 (0.3)	
Mean Day of Enteral Feed Initiation (SD)	2.6 (5.5)	2.3 (5)	0.784
Hyperglycemia Days 2–8 [days]			0.918
Mean (SD)	2.2 (2.6)	2.3 (2.8)	
Hypertriglyceridemia Days 2–8 [days]			0.289
Mean (SD)	0.3 (0.6)	0.2 (0.6)	
Hyperbilirubinemia Days 2–8 [days]			0.43
Mean (SD)	0.3 (1.2)	0.1 (0.5)	

Table S3. Demographics & Inpatient Nutrition by Missing 1 Year Bayley Status.

	Missing (N = 41)	Present (N = 46)	*p*-value
Gestational Age [weeks]			0.563
Mean (SD)	27 (2.4)	27.3 (2.7)	
Birth Weight [kg]			0.92
Mean (SD)	0.9 (0.3)	0.9 (0.3)	
Sex			> 0.99
Female	20 (48.8%)	23 (50%)	
Male	21 (51.2%)	23 (50%)	
Race			0.035*
Asian	4 (9.8%)	3 (6.5%)	
Black/African American	7 (17.1%)	2 (4.3%)	
White/Caucasian	20 (48.8%)	36 (78.3%)	
More Than One Race	2 (4.9%)	0 (0%)	
Other/Unknown	8 (19.5%)	5 (10.9%)	
White Race Status			0.008
White	20 (48.8%)	36 (78.3%)	
Non-White	21 (51.2%)	10 (21.7%)	
Ethnicity			0.481*
Hispanic or Latino	7 (17.1%)	4 (8.7%)	
Not Hispanic or Latino	26 (63.4%)	34 (73.9%)	
Unknown/Not Reported	8 (19.5%)	8 (17.4%)	
Maternal Age [years]			0.033
Mean (SD)	29.2 (6.9)	32 (3.9)	
Antenatal Corticosteroid Use			0.331*
Yes	35 (85.4%)	43 (93.5%)	
No	4 (9.8%)	3 (6.5%)	
Unknown	2 (4.9%)	0 (0%)	
SGA			0.62*
Yes	1 (2.6%)	3 (6.8%)	
No	37 (97.4%)	41 (93.2%)	
Twin Status			0.292
Twin	13 (31.7%)	9 (19.6%)	
Not A Twin	28 (68.3%)	37 (80.4%)	
Apgar Score at 5 Min			0.775
Mean (SD)	7 (1.8)	6.9 (1.9)	
Antibiotic Use (Days 1-7) [days]			0.617
Mean (SD)	2.3 (1.7)	2.6 (2)	
SNAPPE II Scores			0.383
Mean (SD)	22.7 (19.9)	27.1 (26.2)	
Nec Status			0.704*
Yes	4 (9.8%)	3 (6.7%)	
No	37 (90.2%)	42 (93.3%)	
Chronic Lung Disease or BPD at 36 Weeks			0.65
Yes	21 (60%)	31 (67.4%)	
No	14 (40%)	15 (32.6%)	
IVH Status			0.154*
None/Grade 1	37 (90.2%)	36 (78.3%)	

Grade 2/3/4	4 (9.8%)	10 (21.7%)	
ROP Status			> 0.99
None/Stage 0/1	28 (80%)	35 (77.8%)	
Stage 2/3	7 (20%)	10 (22.2%)	
Average Intake (Total Enteral + Parenteral) Days 2–8 [kcal/kg/day]			0.946
Mean (SD)	95.8 (16.2)	96 (15.6)	
Average Protein Intake (Total Enteral + Parenteral) Days 2–8 [g/kg/day]			0.348
Mean (SD)	4.2 (0.3)	4.1 (0.5)	
Average Enteral Intake Days 2–8 [kcal/kg/day]			0.22
Mean (SD)	25.4 (19.8)	20.5 (17.6)	
Average Enteral Protein Intake Days 2–8 [g/kg/day]			0.246
Mean (SD)	0.8 (0.7)	0.6 (0.7)	
Average Parenteral Intake Days 2–8 [kcal/kg/day]			0.07
Mean (SD)	70.4 (11.9)	75.6 (14.6)	
Average Parenteral Protein Intake Days 2–8 [g/kg/day]			0.309
Mean (SD)	3.5 (0.4)	3.6 (0.5)	
Kcal Deficit Over Stay (120 kcal/day goal)			0.424
Mean (SD)	50.9 (672.6)	166.7 (670.8)	
Protein Deficit Over Stay (4 g/day goal)			0.147
Mean (SD)	−9.9 (18.3)	-3 (25.6)	
Mean Day of Enteral Feed Initiation	1.9 (1.5)	3.1 (7.1)	0.242
Insulin Use Days 2–8 [days]			0.601
Mean (SD)	1.2 (2.3)	1.5 (2.3)	
Hyperglycemia Days 2–8 [days]			0.759
Mean (SD)	2.2 (2.6)	2.3 (2.7)	
Hypertriglyceridemia Days 2–8 [days]			0.392
Mean (SD)	0.2 (0.5)	0.3 (0.7)	
Hyperbilirubinemia Days 2–8 [days]			0.983
Mean (SD)	0.2 (1.1)	0.2 (0.9)	

Table S4. Demographics & Inpatient Nutrition by Missing 2 Year Bayley Status.

	**Missing (N = 58)**	**Present (N = 29)**	***p*-value**
Gestational Age [weeks]			0.711
Mean (SD)	27.1 (2.5)	27.3 (2.7)	
Birth Weight [kg]			0.58
Mean (SD)	1 (0.3)	0.9 (0.3)	
Sex			> 0.99
Female	29 (50%)	14 (48.3%)	
Male	29 (50%)	15 (51.7%)	
Race			0.288*
Asian	6 (10.3%)	1 (3.4%)	
Black/African American	8 (13.8%)	1 (3.4%)	
White/Caucasian	33 (56.9%)	23 (79.3%)	
More Than One Race	2 (3.4%)	0 (0%)	
Other/Unknown	9 (15.5%)	4 (13.8%)	
White Race Status			0.069
White	33 (56.9%)	23 (79.3%)	
Non-White	25 (43.1%)	6 (20.7%)	
Ethnicity			0.646*
Hispanic or Latino	8 (13.8%)	3 (10.3%)	
Not Hispanic or Latino	41 (70.7%)	19 (65.5%)	
Unknown/Not Reported	9 (15.5%)	7 (24.1%)	
Maternal Age [years]			0.061
Mean (SD)	30 (6.1)	32.1 (4.2)	
Antenatal Corticosteroid Use			0.717*
Yes	52 (89.7%)	26 (89.7%)	
No	4 (6.9%)	3 (10.3%)	
Unknown	2 (3.4%)	0 (0%)	
SGA			0.102*
Yes	1 (1.8%)	3 (11.1%)	
No	54 (98.2%)	24 (88.9%)	
Twin Status			0.337
Twin	17 (29.3%)	5 (17.2%)	
Not A Twin	41 (70.7%)	24 (82.8%)	
Apgar Score at 5 Min			0.015
Mean (SD)	2 (3.4)	6 (20.7)	
Antibiotic Use (Days 1-7) [days]			0.818
Mean (SD)	2.5 (2)	2.4 (1.5)	
SNAPPE II Scores			0.223
Mean (SD)	22.6 (20.3)	29.9 (28.4)	
Nec Status			> 0.99*
Yes	5 (8.6%)	2 (7.1%)	
No	53 (91.4%)	26 (92.9%)	
Chronic Lung Disease or BPD at 36 Weeks			0.363
Yes	31 (59.6%)	21 (72.4%)	
No	21 (40.4%)	8 (27.6%)	
IVH Status			0.606
None/Grade 1	50 (86.2%)	23 (79.3%)	

Grade 2/3/4	8 (13.8%)	6 (20.7%)	
ROP Status			0.797
None/Stage 0/1	40 (76.9%)	23 (82.1%)	
Stage 2/3	12 (23.1%)	5 (17.9%)	
Average Intake (Total Enteral + Parenteral) Days 2–8 [kcal/kg/day]			0.612
Mean (SD)	95.3 (15.5)	97.2 (16.4)	
Average Protein Intake (Total Enteral + Parenteral) Days 2–8 [g/kg/day]			0.743
Mean (SD)	4.2 (0.4)	4.2 (0.2)	
Average Enteral Intake Days 2–8 [kcal/kg/day]			0.384
Mean (SD)	24 (19.2)	20.4 (17.7)	
Average Enteral Protein Intake Days 2–8 [g/kg/day]			0.475
Mean (SD)	0.7 (0.7)	0.6 (0.7)	
Average Parenteral Intake Days 2–8 [kcal/kg/day]			0.089
Mean (SD)	71.3 (12.9)	76.8 (14.5)	
Average Parenteral Protein Intake Days 2–8 [g/kg/day]			0.084
Mean (SD)	3.5 (0.4)	3.7 (0.4)	
Kcal Deficit Over Stay (120 kcal/day goal)			0.688
Mean (SD)	133.3 (655)	69.8 (709.7)	
Protein Deficit Over Stay (4 g/day goal)			0.934
Mean (SD)	−6.1 (22.5)	−6.6 (23.1)	
Mean Day of Enteral Feed Initiation	2.6 (5.5)	2.5 (5.1)	0.954
Insulin Use Days 2–8 [days]			0.717
Mean (SD)	1.3 (2.3)	1.5 (2.3)	
Hyperglycemia Days 2–8 [days]			0.72
Mean (SD)	2.2 (2.6)	2.4 (2.8)	
Hypertriglyceridemia Days 2–8 [days]			0.479
Mean (SD)	0.3 (0.6)	0.4 (0.6)	
Hyperbilirubinemia Days 2–8 [days]			0.502
Mean (SD)	0.1 (0.9)	0.3 (1.1)	

Sensitivity Analysis - Attrition Bias (Tables S5–S13)

Table S5. ERP/VEP Analysis - Model 1 (Adjusted for Sex, Gestational Age, and Age at Test [VEP/ERP Only]).

VEP - P100 Latency	Control (N = 18) | Intervention (N = 15)		
Study Arm: Intervention [ref. Control]			
Sex: Male [ref. Female]			
Gestation Age [weeks]			
Age at ERP/VEP Test [days]			
	**Effect Estimate**	**95% CI**	***p*-value**
VEP - P100 Latency			
Study Arm: Intervention [ref. Control]	34.61	(11.97, 57.25)	0.004
Sex: Male [ref. Female]	−6.83	(−29.17, 15.5)	0.536
Gestation Age [weeks]	0.77	(−4.23, 5.77)	0.755
Age at ERP/VEP Test [days]	−0.06	(−0.93, 0.81)	0.881

Table S6. ERP/VEP Analysis - Model 2 (Adjusted for Sex, Gestational Age, Kcal/kg from Feeds, and Age at Test [VEP/ERP Only]).

VEP - P100 Latency	Control (N = 18) ∣ Intervention (N = 15)		
Study Arm: Intervention [ref. Control]			
Sex: Male [ref. Female]			
Gestation Age [weeks]			
Average Enteral Intake Days 2–8 [kcal/kg/day]			
Age at ERP/VEP Test [days]			
	**Effect Estimate**	**95% CI**	***p*-value**
VEP - P100 Latency			
Study Arm: Intervention [ref. Control]	22.43	(−4.81, 49.68)	0.103
Sex: Male [ref. Female]	−15.4	(−40.17, 9.37)	0.213
Gestation Age [weeks]	4.14	(−2.58, 10.87)	0.217
Average Enteral Intake Days 2–8 [kcal/kg/day]	−0.66	(−1.52, 0.2)	0.128
Age at ERP/VEP Test [days]	0.08	(−0.8, 0.96)	0.854

Table S7. ERP/VEP Analysis - Model 3: Multivariable Condition by Specific Intake Days 2–8.

VEP - P100 Latency		Control (N = 18) ∣ Intervention (N = 15)	
Average Total Intake [kcal/kg/day]			
Sex: Male [ref. Female]			
Gestation Age [weeks]			
Age at ERP/VEP Test [days]			
VEP - P100 Latency		Control (N = 18) ∣ Intervention (N = 15)	
Average Total Intake [kcal/kg/day]			
Sex: Male [ref. Female]			
Gestation Age [weeks]			
Age at ERP/VEP Test [days]			
VEP - P100 Latency		Control (N = 18) ∣ Intervention (N = 15)	
Average Total Intake [kcal/kg/day]			
Sex: Male [ref. Female]			
Gestation Age [weeks]			
Age at ERP/VEP Test [days]			
VEP - P100 Latency		Control (N = 18) ∣ Intervention (N = 15)	
Average Total Intake [kcal/kg/day]			
Sex: Male [ref. Female]			
Gestation Age [weeks]			
Age at ERP/VEP Test [days]			
	**Effect Estimate**	**95% CI**	***p*-value**
VEP - P100 Latency			
Average Total Intake [kcal/kg/day]	−0.4	(−1.83, 1.02)	0.567
Sex: Male [ref. Female]	3.07	(−22.19, 28.33)	0.805
Gestation Age [weeks]	−0.25	(−7.97, 7.46)	0.947
Age at ERP/VEP Test [days]	−0.22	(−1.23, 0.8)	0.661
VEP - P100 Latency			
Average Total Intake [kcal/kg/day]	−45.54	(−99.38, 8.3)	0.094
Sex: Male [ref. Female]	−11.85	(−42.43, 18.73)	0.434
Gestation Age [weeks]	2	(−4.9, 8.91)	0.557
Age at ERP/VEP Test [days]	0.15	(−0.92, 1.23)	0.772
VEP - P100 Latency			
Average Total Intake [kcal/kg/day]	−1.07	(−1.79, −0.35)	0.005
Sex: Male [ref. Female]	−15.87	(−41.4, 9.65)	0.213
Gestation Age [weeks]	5.34	(−1.43, 12.11)	0.213
Age at ERP/VEP Test [days]	0.09	(−0.81, 1)	0.832
VEP - P100 Latency			
Average Total Intake [kcal/kg/day]	−26.26	(−46.91, −5.62)	0.015
Sex: Male [ref. Female]	−19.86	(−49.28, 9.57)	0.178
Gestation Age [weeks]	5.39	(−2.12, 12.9)	0.153
Age at ERP/VEP Test [days]	0.1	(−0.85, 1.05)	0.83
Average Total Intake [kcal/kg/day]	1.01	(0.24, 1.78)	0.012
Sex: Male [ref. Female]	−10.91	(−35.97, 14.16)	0.38
Gestation Age [weeks]	1.1	(−4.29, 6.49)	0.679

Age at ERP/VEP Test [days]	−0.06	(−0.97, 0.85)	0.892
Average Total Intake [kcal/kg/day]	46.54	(11.72, 81.36)	0.011
Sex: Male [ref. Female]	-13.94	(−39.99, 12.11)	0.282
Gestation Age [weeks]	4.01	(−2.54, 10.56)	0.22
Age at ERP/VEP Test [days]	-0.08	(−0.98, 0.82)	0.86

Table S8. Bayley Score - Year 1 - Model 1 (Adjusted for Sex, Gestational Age, and Age at Test [VEP/ERP Only]).

<table>
<tr><td></td><td></td><td></td><td></td></tr>
<tr><td colspan="2">Bayley - 1 year: Cognitive</td><td colspan="2">Control (N = 23) | Intervention (N = 23)</td></tr>
<tr><td colspan="2">Study Arm: Intervention [ref. Control]</td><td colspan="2"></td></tr>
<tr><td colspan="2">Sex: Male [ref. Female]</td><td colspan="2"></td></tr>
<tr><td colspan="2">Gestation Age [weeks]</td><td colspan="2"></td></tr>
<tr><td colspan="2">Bayley - 1 year: Cognitive</td><td colspan="2">Control (N = 23) | Intervention (N = 23)</td></tr>
<tr><td colspan="2">Study Arm: Intervention [ref. Control]</td><td colspan="2"></td></tr>
<tr><td colspan="2">Sex: Male [ref. Female]</td><td colspan="2"></td></tr>
<tr><td colspan="2">Gestation Age [weeks]</td><td colspan="2"></td></tr>
<tr><td colspan="2">Bayley - 1 year: Cognitive</td><td colspan="2">Control (N = 23) | Intervention (N = 23)</td></tr>
<tr><td colspan="2">Study Arm: Intervention [ref. Control]</td><td colspan="2"></td></tr>
<tr><td colspan="2">Sex: Male [ref. Female]</td><td colspan="2"></td></tr>
<tr><td colspan="2">Gestation Age [weeks]</td><td colspan="2"></td></tr>
<tr><td></td><td>Effect Estimate</td><td>95% CI</td><td>p-value</td></tr>
<tr><td>Bayley - 1 year: Cognitive</td><td></td><td></td><td></td></tr>
<tr><td>Study Arm: Intervention [ref. Control]</td><td>6.5</td><td>(−2.41, 15.42)</td><td>0.148</td></tr>
<tr><td>Sex: Male [ref. Female]</td><td>9.52</td><td>(0.55, 18.49)</td><td>0.038</td></tr>
<tr><td>Gestation Age [weeks]</td><td>2.99</td><td>(1.38, 4.61)</td><td>< 0.001</td></tr>
<tr><td>Bayley - 1 year: Cognitive</td><td></td><td></td><td></td></tr>
<tr><td>Study Arm: Intervention [ref. Control]</td><td>3.14</td><td>(−6.17, 12.44)</td><td>0.5</td></tr>
<tr><td>Sex: Male [ref. Female]</td><td>3.85</td><td>(−5.51, 13.22)</td><td>0.411</td></tr>
<tr><td>Gestation Age [weeks]</td><td>4.25</td><td>(2.53, 5.96)</td><td>< 0.001</td></tr>
<tr><td>Bayley - 1 year: Cognitive</td><td></td><td></td><td></td></tr>
<tr><td>Study Arm: Intervention [ref. Control]</td><td>3.47</td><td>(−6.36, 13.3)</td><td>0.479</td></tr>
<tr><td>Sex: Male [ref. Female]</td><td>7.27</td><td>(−2.67, 17.2)</td><td>0.147</td></tr>
<tr><td>Gestation Age [weeks]</td><td>4.3</td><td>(2.52, 6.07)</td><td>< 0.001</td></tr>
</table>

Table S9. Bayley Score - Year 1 - Model 2 (Adjusted for Sex, Gestational Age, Kcal/kg from Feeds, and Age at Test [VEP/ERP Only]).

Bayley - 1 year: Cognitive		Control (N = 23) ǀ Intervention (N = 23)	
Study Arm: Intervention [ref. Control]			
Sex: Male [ref. Female]			
Gestation Age [weeks]			
Average Enteral Intake Days 2–8 [kcal/kg/day]			
Bayley - 1 year: Cognitive		Control (N = 23) ǀ Intervention (N = 23)	
Study Arm: Intervention [ref. Control]			
Sex: Male [ref. Female]			
Gestation Age [weeks]			
Average Enteral Intake Days 2–8 [kcal/kg/day]			
Bayley - 1 year: Cognitive		Control (N = 23) ǀ Intervention (N = 23)	
Study Arm: Intervention [ref. Control]			
Sex: Male [ref. Female]			
Gestation Age [weeks]			
Average Enteral Intake Days 2–8 [kcal/kg/day]			
	**Effect Estimate**	**95% CI**	***p*-value**
Bayley - 1 year: Cognitive			
Study Arm: Intervention [ref. Control]	6.44	(−3.14, 16.03)	0.181
Sex: Male [ref. Female]	9.53	(0.41, 18.65)	0.041
Gestation Age [weeks]	3.02	(0.91, 5.13)	0.006
Average Enteral Intake Days 2–8 [kcal/kg/day]	−0.01	(−0.34, 0.33)	0.97
Bayley - 1 year: Cognitive			
Study Arm: Intervention [ref. Control]	1.52	(−8.4, 11.44)	0.758
Sex: Male [ref. Female]	4.13	(−5.27, 13.53)	0.379
Gestation Age [weeks]	4.88	(2.71, 7.05)	< 0.001
Average Enteral Intake Days 2–8 [kcal/kg/day]	−0.16	(−0.5, 0.18)	0.34
Bayley - 1 year: Cognitive			
Study Arm: Intervention [ref. Control]	2.09	(−8.39, 12.57)	0.689
Sex: Male [ref. Female]	7.57	(−2.44, 17.59)	0.134
Gestation Age [weeks]	4.88	(2.56, 7.19)	< 0.001
Average Enteral Intake Days 2–8 [kcal/kg/day]	−0.15	(−0.51, 0.22)	0.428

Table S10. Bayley Score - Year 1 - Model 3 - Multivariable Condition by Specific Intake Days 2–8.

Bayley - 1 Year: Cognitive	Control (N = 23) ǀ Intervention (N = 23)
Average Total Intake [kcal/kg/day]	
Sex: Male [ref. Female]	
Gestation Age [weeks]	
Bayley - 1 Year: Cognitive	Control (N = 23) ǀ Intervention (N = 23)
Average Total Intake [kcal/kg/day]	
Sex: Male [ref. Female]	
Gestation Age [weeks]	
Bayley - 1 Year: Cognitive	Control (N = 23) ǀ Intervention (N = 23)
Average Total Intake [kcal/kg/day]	
Sex: Male [ref. Female]	
Gestation Age [weeks]	
Bayley - 1 Year: Cognitive	Control (N = 23) ǀ Intervention (N = 23)
Average Total Intake [kcal/kg/day]	
Sex: Male [ref. Female]	
Gestation Age [weeks]	
Bayley - 1 Year: Cognitive	Control (N = 23) ǀ Intervention (N = 23)
Average Total Intake [kcal/kg/day]	
Sex: Male [ref. Female]	
Gestation Age [weeks]	
Bayley - 1 Year: Cognitive	Control (N = 23) ǀ Intervention (N = 23)
Average Total Intake [kcal/kg/day]	
Sex: Male [ref. Female]	
Gestation Age [weeks]	
Bayley - 1 Year: Cognitive	Control (N = 23) ǀ Intervention (N = 23)
Average Total Intake [kcal/kg/day]	
Sex: Male [ref. Female]	
Gestation Age [weeks]	
Bayley - 1 Year: Cognitive	Control (N = 23) ǀ Intervention (N = 23)
Average Total Intake [kcal/kg/day]	
Sex: Male [ref. Female]	
Gestation Age [weeks]	
Bayley - 1 Year: Cognitive	Control (N = 23) ǀ Intervention (N = 23)
Average Total Intake [kcal/kg/day]	
Sex: Male [ref. Female]	
Gestation Age [weeks]	
Bayley - 1 Year: Cognitive	Control (N = 23) ǀ Intervention (N = 23)
Average Total Intake [kcal/kg/day]	
Sex: Male [ref. Female]	
Gestation Age [weeks]	
Bayley - 1 Year: Cognitive	Control (N = 23) ǀ Intervention (N = 23)
Average Total Intake [kcal/kg/day]	
Sex: Male [ref. Female]	
Gestation Age [weeks]	

Bayley - 1 Year: Cognitive		Control (N = 23) ∣ Intervention (N = 23)	
Average Total Intake [kcal/kg/day]			
Sex: Male [ref. Female]			
Gestation Age [weeks]			
	**Effect Estimate**	**95% CI**	***p*-value**
Bayley - 1 Year: Cognitive			
Average Total Intake [kcal/kg/day]	0.16	(−0.15, 0.48)	0.309
Sex: Male [ref. Female]	9.07	(−0.02, 18.13)	0.05
Gestation Age [weeks]	2.47	(0.5, 4.44)	0.015
Bayley - 1 Year: Language			
Average Total Intake [kcal/kg/day]	−0.07	(−0.4, 0.26)	0.668
Sex: Male [ref. Female]	3.26	(−6.08, 12.59)	0.485
Gestation Age [weeks]	4.4	(2.25, 6.56)	< 0.001
Bayley - 1 Year: Cognitive			
Average Total Intake [kcal/kg/day]	−0.03	(−0.38, 0.32)	0.862
Sex: Male [ref. Female]	6.85	(−3.1, 16.79)	0.172
Gestation Age [weeks]	4.4	(2.25, 6.56)	< 0.001
Bayley - 1 Year: Cognitive			
Average Total Intake [kcal/kg/day]	−0.84	(−8.97, 7.29)	0.836
Sex: Male [ref. Female]	8.85	(−0.34, 18.03)	0.059
Gestation Age [weeks]	3.04	(1.38, 4.71)	< 0.001
Bayley - 1 Year: Cognitive			
Average Total Intake [kcal/kg/day]	−4.3	(−12.46, 3.86)	0.293
Sex: Male [ref. Female]	3.03	(−6.2, 12.25)	0.511
Gestation Age [weeks]	4.28	(2.58, 5.98)	< 0.001
Bayley - 1 Year: Cognitive			
Average Total Intake [kcal/kg/day]	−3.71	(−12.51, 5.09)	0.399
Sex: Male [ref. Female]	6.61	(−3.27, 16.48)	0.184
Gestation Age [weeks]	4.37	(2.59, 6.16)	< 0.001
Bayley - 1 Year: Cognitive			
Average Total Intake [kcal/kg/day]	−0.08	(−0.4, 0.24)	0.611
Sex: Male [ref. Female]	9.15	(−0.04, 18.34)	0.051
Gestation Age [weeks]	3.34	(−0.4, 6.16)	0.002
Bayley - 1 Year: Cognitive			
Average Total Intake [kcal/kg/day]	−0.18	(−0.5, 0.14)	0.256
Sex: Male [ref. Female]	3.97	(−5.26, 13.2)	0.389
Gestation Age [weeks]	4.94	(2.83, 7.05)	< 0.001
Bayley - 1 Year: Cognitive			
Average Total Intake [kcal/kg/day]	−0.17	(−0.51, 0.17)	0.322
Sex: Male [ref. Female]	7.42	(−2.46, 17.29)	0.137
Gestation Age [weeks]	4.98	(2.75, 7.21)	< 0.001
Bayley - 1 Year: Cognitive			
Average Total Intake [kcal/kg/day]	−2.75	(−11.13, 5.63)	0.511
Sex: Male [ref. Female]	9.07	(−0.06, 18.21)	0.052
Gestation Age [weeks]	3.48	(1.33, 5.62)	0.002
Bayley - 1 Year: Cognitive			
Average Total Intake [kcal/kg/day]	−3.76	(−12.22, 4.7)	0.374

Sex: Male [ref. Female]	3.67	(−5.59, 12.92)	0.428
Gestation Age [weeks]	4.85	(2.64, 7.06)	< 0.001
Bayley - 1 Year: Cognitive			
Average Total Intake [kcal/kg/day]	−5.01	(−14.07, 4.05)	0.27
Sex: Male [ref. Female]	7.18	(−2.63, 16.98)	0.147
Gestation Age [weeks]	5.12	(2.82, 7.43)	< 0.001
Bayley - 1 Year: Cognitive			
Average Total Intake [kcal/kg/day]	0.23	(−0.07, 0.53)	0.135
Sex: Male [ref. Female]	9.82	(0.83, 18.82)	0.033
Gestation Age [weeks]	3.13	(1.52, 4.75)	< 0.001
Bayley - 1 Year: Cognitive			
Average Total Intake [kcal/kg/day]	0.11	(−0.21, 0.43)	0.482
Sex: Male [ref. Female]	4	(−5.42, 13.42)	0.396
Gestation Age [weeks]	4.31	(2.58, 6.04)	< 0.001
Bayley - 1 Year: Cognitive			
Average Total Intake [kcal/kg/day]	0.13	(−0.2, 0.46)	0.431
Sex: Male [ref. Female]	7.45	(−2.51, 17.42)	0.139
Gestation Age [weeks]	4.37	(2.59, 6.16)	< 0.001
Bayley - 1 Year: Cognitive			
Average Total Intake [kcal/kg/day]	1.66	(−8.45, 11.77)	0.741
Sex: Male [ref. Female]	9.13	(−0.13, 18.39)	0.053
Gestation Age [weeks]	3.19	(1.25, 5.14)	0.002
Bayley - 1 Year: Cognitive			
Average Total Intake [kcal/kg/day]	−2	(−12.32, 8.31)	0.697
Sex: Male [ref. Female]	3.11	(−6.33, 12.55)	0.51
Gestation Age [weeks]	4.01	(1.97, 6.04)	< 0.001
Bayley - 1 Year: Cognitive			
Average Total Intake [kcal/kg/day]	−0.82	(−11.86, 10.22)	0.881
Sex: Male [ref. Female]	6.77	(−3.27, 16.82)	0.181
Gestation Age [weeks]	4.22	(2.11, 6.33)	< 0.001

Table S11. Bayley Score - Year 2 - Model 1 (Adjusted for Sex, Gestational Age, and Age at Test [VEP/ERP Only]).

Bayley - 2 year: Cognitive		Control (N = 13) ∣ Intervention (N = 16)	
Study Arm: Intervention [ref. Control]			
Sex: Male [ref. Female]			
Gestation Age [weeks]			
Bayley - 2 year: Cognitive		Control (N = 13) ∣ Intervention (N = 16)	
Study Arm: Intervention [ref. Control]			
Sex: Male [ref. Female]			
Gestation Age [weeks]			
Bayley - 2 year: Cognitive		Control (N = 13) ∣ Intervention (N = 16)	
Study Arm: Intervention [ref. Control]			
Sex: Male [ref. Female]			
Gestation Age [weeks]			
	**Effect Estimate**	**95% CI**	***p*-value**
Bayley - 2 year: Cognitive			
Study Arm: Intervention [ref. Control]	0.54	(−14.64, 15.73)	0.942
Sex: Male [ref. Female]	−2.66	(−18.75, 13.42)	0.736
Gestation Age [weeks]	3.44	(0.44, 6.45)	0.027
Bayley - 2 year: Language			
Study Arm: Intervention [ref. Control]	−1.33	(−17.39, 14.72)	0.865
Sex: Male [ref. Female]	−2.36	(−19.45, 14.74)	0.778
Gestation Age [weeks]	3.18	(0.03, 6.33)	0.048
Bayley - 2 year: Motor			
Study Arm: Intervention [ref. Control]	−4.43	(−17.71, 8.85)	0.499
Sex: Male [ref. Female]	−2.08	(−16.42, 12.25)	0.767
Gestation Age [weeks]	4.81	(2.17, 7.44)	< 0.001

Table S12. Bayley Score - Year 2 - Model 2 (Adjusted for Sex, Gestational Age, Kcal/kg from Feeds, and Age at Test [VEP/ERP Only]).

Bayley - 2 year: Cognitive		Control (N = 13) ǀ Intervention (N = 16)	
Study Arm: Intervention [ref. Control]			
Sex: Male [ref. Female]			
Gestation Age [weeks]			
Average Enteral Intake Days 2–8 [kcal/kg/day]			
Bayley - 2 year: Cognitive		Control (N = 13) ǀ Intervention (N = 16)	
Study Arm: Intervention [ref. Control]			
Sex: Male [ref. Female]			
Gestation Age [weeks]			
Average Enteral Intake Days 2–8 [kcal/kg/day]			
Bayley - 2 year: Cognitive		Control (N = 13) ǀ Intervention (N = 16)	
Study Arm: Intervention [ref. Control]			
Sex: Male [ref. Female]			
Gestation Age [weeks]			
Average Enteral Intake Days 2–8 [kcal/kg/day]			
	**Effect Estimate**	**95% CI**	***p*-value**
Bayley - 2 year: Cognitive			
Study Arm: Intervention [ref. Control]	2.13	(−15.43, 19.69)	0.804
Sex: Male [ref. Female]	−2.69	(−19.1, 13.72)	0.738
Gestation Age [weeks]	2.71	(−2.18, 7.6)	0.263
Average Enteral Intake Days 2–8 [kcal/kg/day]	0.14	(−0.59, 0.87)	0.695
Bayley - 2 year: Cognitive			
Study Arm: Intervention [ref. Control]	1.77	(−16.64, 20.18)	0.844
Sex: Male [ref. Female]	−2.33	(−19.64, 14.98)	0.783
Gestation Age [weeks]	1.81	(−3.18, 6.79)	0.46
Average Enteral Intake Days 2–8 [kcal/kg/day]	0.27	(−0.48, 1.03)	0.465
Bayley - 2 year: Cognitive			
Study Arm: Intervention [ref. Control]	−4.15	(−19.51, 11.22)	0.583
Sex: Male [ref. Female]	−2.09	(−16.74, 12.57)	0.771
Gestation Age [weeks]	4.67	(0.36, 8.98)	0.035
Average Enteral Intake Days 2–8 [kcal/kg/day]	0.03	(−0.63, 0.68)	0.936

Table S13. Bayley Score - Year 2 - Model 3: Multivariable Condition by Specific Intake Days 2–8.

Bayley - 2 Year: Cognitive	Control (N = 13) ǀ Intervention (N = 16)
Average Total Intake [kcal/kg/day]	
Sex: Male [ref. Female]	
Gestation Age [weeks]	
Bayley - 2 Year: Cognitive	Control (N = 13) ǀ Intervention (N = 16)
Average Total Intake [kcal/kg/day]	
Sex: Male [ref. Female]	
Gestation Age [weeks]	
Bayley - 2 Year: Cognitive	Control (N = 13) ǀ Intervention (N = 16)
Average Total Intake [kcal/kg/day]	
Sex: Male [ref. Female]	
Gestation Age [weeks]	
Bayley - 2 Year: Cognitive	Control (N = 13) ǀ Intervention (N = 16)
Average Total Intake [kcal/kg/day]	
Sex: Male [ref. Female]	
Gestation Age [weeks]	
Bayley - 2 Year: Cognitive	Control (N = 13) ǀ Intervention (N = 16)
Average Total Intake [kcal/kg/day]	
Sex: Male [ref. Female]	
Gestation Age [weeks]	
Bayley - 2 Year: Cognitive	Control (N = 13) ǀ Intervention (N = 16)
Average Total Intake [kcal/kg/day]	
Sex: Male [ref. Female]	
Gestation Age [weeks]	
Bayley - 2 Year: Cognitive	Control (N = 13) ǀ Intervention (N = 16)
Average Total Intake [kcal/kg/day]	
Sex: Male [ref. Female]	
Gestation Age [weeks]	
Bayley - 2 Year: Cognitive	Control (N = 13) ǀ Intervention (N = 16)
Average Total Intake [kcal/kg/day]	
Sex: Male [ref. Female]	
Gestation Age [weeks]	
Bayley - 2 Year: Cognitive	Control (N = 13) ǀ Intervention (N = 16)
Average Total Intake [kcal/kg/day]	
Sex: Male [ref. Female]	
Gestation Age [weeks]	
Bayley - 2 Year: Cognitive	Control (N = 13) ǀ Intervention (N = 16)
Average Total Intake [kcal/kg/day]	
Sex: Male [ref. Female]	
Gestation Age [weeks]	
Bayley - 2 Year: Cognitive	Control (N = 13) ǀ Intervention (N = 16)
Average Total Intake [kcal/kg/day]	
Sex: Male [ref. Female]	
Gestation Age [weeks]	
Bayley - 2 Year: Cognitive	Control (N = 13) ǀ Intervention (N = 16)
Average Total Intake [kcal/kg/day]	
Sex: Male [ref. Female]	
Gestation Age [weeks]	
Bayley - 2 Year: Cognitive	Control (N = 13) ǀ Intervention (N = 16)
Average Total Intake [kcal/kg/day]	
Sex: Male [ref. Female]	
Gestation Age [weeks]	

Bayley - 2 Year: Cognitive	Control (N = 13) ∣ Intervention (N = 16)
Average Total Intake [kcal/kg/day]	
Sex: Male [ref. Female]	
Gestation Age [weeks]	
Bayley - 2 Year: Cognitive	Control (N = 13) ∣ Intervention (N = 16)
Average Total Intake [kcal/kg/day]	
Sex: Male [ref. Female]	
Gestation Age [weeks]	
Bayley - 2 Year: Cognitive	Control (N = 13) ∣ Intervention (N = 16)
Average Total Intake [kcal/kg/day]	
Sex: Male [ref. Female]	
Gestation Age [weeks]	
Bayley - 2 Year: Cognitive	Control (N = 13) ∣ Intervention (N = 16)
Average Total Intake [kcal/kg/day]	
Sex: Male [ref. Female]	
Gestation Age [weeks]	
Bayley - 2 Year: Cognitive	Control (N = 13) ∣ Intervention (N = 16)
Average Total Intake [kcal/kg/day]	
Sex: Male [ref. Female]	
Gestation Age [weeks]	

	Effect Estimate	95% CI	*p*-value
Bayley - 2 Year: Cognitive			
Average Total Intake [kcal/kg/day]	−0.2	(−0.94, 0.53)	0.571
Sex: Male [ref. Female]	−3.19	(−18.77, 12.39)	0.676
Gestation Age [weeks]	4.59	(−0.36, 9.54)	0.068
Bayley - 2 Year: Cognitive			
Average Total Intake [kcal/kg/day]	−0.07	(−0.9, 0.76)	0.865
Sex: Male [ref. Female]	−2.29	(−19.2, 14.62)	0.782
Gestation Age [weeks]	3.49	(−1.77, 8.75)	0.183
Bayley - 2 Year: Cognitive			
Average Total Intake [kcal/kg/day]	−0.16	(−0.81, 0.5)	0.626
Sex: Male [ref. Female]	−1.22	(−15.22, 12.78)	0.859
Gestation Age [weeks]	5.46	(1.1, 9.82)	0.016
Bayley - 2 Year: Cognitive			
Average Total Intake [kcal/kg/day]	8.59	(−28.73, 45.92)	0.639
Sex: Male [ref. Female]	−3.55	(−19.44, 12.34)	0.649
Gestation Age [weeks]	3.05	(−0.39, 6.49)	0.08
Bayley - 2 Year: Cognitive			
Average Total Intake [kcal/kg/day]	6.17	(−33.51, 45.85)	0.751
Sex: Male [ref. Female]	−2.45	(−19.21, 14.32)	0.765
Gestation Age [weeks]	2.87	(−0.72, 6.45)	0.112
Bayley - 2 Year: Cognitive			
Average Total Intake [kcal/kg/day]	13.15	(−20.17, 46.47)	0.424
Sex: Male [ref. Female]	−1.98	(−16.07, 12.11)	0.775
Gestation Age [weeks]	3.98	(0.94, 7.02)	0.012
Bayley - 2 Year: Cognitive			
Average Total Intake [kcal/kg/day]	0.1	(−0.53, 0.73)	0.749
Sex: Male [ref. Female]	−3.1	(−18.81, 12.61)	0.687
Gestation Age [weeks]	3.01	(−1.13, 7.15)	0.146
Bayley - 2 Year: Cognitive			
Average Total Intake [kcal/kg/day]	0.24	(−0.41, 0.89)	0.46
Sex: Male [ref. Female]	−2.7	(−19.17, 13.77)	0.737
Gestation Age [weeks]	2.02	(−2.34, 6.38)	0.348
Bayley - 2 Year: Cognitive			

Average Total Intake [kcal/kg/day]	0.11	(−0.46, 0.68)	0.698
Sex: Male [ref. Female]	−1.2	(−15.25, 12.86)	0.862
Gestation Age [weeks]	4.11	(0.4, 7.83)	0.031
Bayley - 2 Year: Cognitive			
Average Total Intake [kcal/kg/day]	2.74	(−11.96, 17.44)	0.704
Sex: Male [ref. Female]	−2.73	(−18.31, 12.85)	0.721
Gestation Age [weeks]	2.98	(−0.94, 6.9)	0.13
Bayley - 2 Year: Cognitive			
Average Total Intake [kcal/kg/day]	3.75	(−11.56, 19.06)	0.617
Sex: Male [ref. Female]	−1.98	(−18.44, 14.49)	0.806
Gestation Age [weeks]	2.46	(−1.72, 6.63)	0.236
Bayley - 2 Year: Cognitive			
Average Total Intake [kcal/kg/day]	1.9	(−11.37, 15.17)	0.771
Sex: Male [ref. Female]	−0.81	(−14.77, 13.15)	0.906
Gestation Age [weeks]	4.28	(0.76, 7.8)	0.019
Bayley - 2 Year: Cognitive			
Average Total Intake [kcal/kg/day]	−0.17	(−0.68, 0.35)	0.509
Sex: Male [ref. Female]	−3.63	(−19.32, 12.06)	0.638
Gestation Age [weeks]	3.6	(0.68, 6.52)	0.018
Bayley - 2 Year: Cognitive			
Average Total Intake [kcal/kg/day]	−0.2	(−0.74, 0.35)	0.462
Sex: Male [ref. Female]	−3.42	(−20.25, 13.4)	0.678
Gestation Age [weeks]	3.21	(0.12, 6.31)	0.042
Bayley - 2 Year: Cognitive			
Average Total Intake [kcal/kg/day]	−0.15	(−0.61, 0.31)	0.51
Sex: Male [ref. Female]	−1.67	(−15.76, 12.41)	0.809
Gestation Age [weeks]	4.72	(2.12, 7.32)	< 0.001
Bayley - 2 Year: Cognitive			
Average Total Intake [kcal/kg/day]	−3.17	(−28.35, 22.01)	0.797
Sex: Male [ref. Female]	−2.68	(−18.31, 12.95)	0.726
Gestation Age [weeks]	3.21	(−0.38, 6.8)	0.078
Bayley - 2 Year: Cognitive			
Average Total Intake [kcal/kg/day]	−8.34	(−34.83, 18.16)	0.522
Sex: Male [ref. Female]	−1.9	(−18.3, 14.51)	0.813
Gestation Age [weeks]	2.39	(−1.52, 6.3)	0.219
Bayley - 2 Year: Cognitive			
Average Total Intake [kcal/kg/day]	−0.59	(−23.28, 22.09)	0.958
Sex: Male [ref. Female]	−0.84	(−14.84, 13.16)	0.903
Gestation Age [weeks]	4.57	(−1.34, 7.8)	0.007
